# Delta-Like Protein 3 Expression in Paired Chemonaive and Chemorelapsed Small Cell Lung Cancer Samples

**DOI:** 10.3389/fmed.2021.734901

**Published:** 2021-10-08

**Authors:** Christiane Kuempers, Tobias Jagomast, Rosemarie Krupar, Finn-Ole Paulsen, Carsten Heidel, Julika Ribbat-Idel, Christian Idel, Bruno Märkl, Martin Anlauf, Sabina Berezowska, Markus Tiemann, Hans Bösmüller, Falko Fend, Barbara Kalsdorf, Sabine Bohnet, Eva Dreyer, Verena Sailer, Jutta Kirfel, Sven Perner

**Affiliations:** ^1^Institute of Pathology, Luebeck, University Hospital Schleswig-Holstein, Luebeck, Germany; ^2^Pathology, Research Center Borstel-Leibniz Lung Center, Borstel, Germany; ^3^Department of Oncology, Hematology and Bone Marrow Transplantation With Division of Pneumology, University Medical Center Hamburg-Eppendorf, Hamburg, Germany; ^4^Department of Otorhinolaryngology, Luebeck, University of Luebeck and University Hospital Schleswig-Holstein, Luebeck, Germany; ^5^Medical Faculty, General Pathology and Molecular Diagnostics, University Augsburg, Augsburg, Germany; ^6^Institute of Pathology, Cytology and Molecular Pathology Limburg, Limburg, Germany; ^7^Department of Laboratory Medicine and Pathology, Institute of Pathology, Lausanne University Hospital and University of Lausanne, Lausanne, Switzerland; ^8^Institute of Pathology, University of Bern, Bern, Switzerland; ^9^Institute for Hematopathology, Hamburg, Germany; ^10^Institute of Pathology and Neuropathology University Hospital Tuebingen, Tuebingen, Germany; ^11^Medical Clinic, Research Center Borstel-Leibniz Lung Center, Borstel, Germany; ^12^Department of Pulmonology, Luebeck, University Hospital Schleswig-Holstein, Luebeck, Germany; ^13^Airway Research Center North (ARCN), Member of the German Center for Lung Research (DZL), Borstel, Germany

**Keywords:** delta-like protein 3, small cell lung cancer, paired, chemonaive, chemorelapsed

## Abstract

Rovalpituzumab tesirine (Rova-T), an antibody-drug conjugate directed against Delta-like protein 3 (DLL3), is under development for patients with small cell lung cancer (SCLC). DLL3 is expressed on the majority of SCLC samples. Because SCLC is rarely biopsied in the course of disease, data regarding DLL3 expression in relapses is not available. The aim of this study was to investigate the expression of DLL3 in chemorelapsed (but untreated with Rova-T) SCLC samples and compare the results with chemonaive counterparts. Two evaluation methods to assess DLL3 expression were explored. Additionally, we assessed if DLL3 expression of chemorelapsed and/or chemonaive samples has prognostic impact and if it correlates with other clinicopathological data. The study included 30 paired SCLC samples, which were stained with an anti DLL3 antibody. DLL3 expression was assessed using tumor proportion score (TPS) and H-score and was categorized as DLL3 low (TPS < 50%, H-score ≤ 150) and DLL3 high (TPS ≥ 50%, H-score > 150). Expression data were correlated with clinicopathological characteristics. Kaplan–Meier curves were used to illustrate overall survival (OS) depending on DLL3 expression in chemonaive and chemorelapsed samples, respectively, and depending on dynamics of expression during course of therapy. DLL3 was expressed in 86.6% chemonaive and 80% chemorelapsed SCLC samples without significant differences between the two groups. However, the extent of expression varied in a substantial proportion of pairs (36.6% with TPS, 43.3% with H-score), defined as a shift from low to high or high to low expression. TPS and H-score provided comparable results. There were no profound correlations with clinicopathological data. Survival analysis revealed a trend toward a more favorable OS in DLL low-expressing chemonaive SCLC (*p* = 0.57) and, in turn, in DLL3 high-expressing chemorelapsed SCLC (*p* = 0.42) as well as in SCLC demonstrating a shift from low to high expression (*p* = 0.56) without being statistically significant. This is the first study to investigate DLL3 expression in a large cohort of rare paired chemonaive-chemorelapsed SCLC specimens. Comparative analysis revealed that DLL3 expression was not stable during the course of therapy, suggesting therapy-based alterations. Unlike in chemonaive samples, a high DLL3 expression in chemorelapsed samples indicated a trend for a more favorable prognosis. Our results highlight the importance to investigate DLL3 in latest chemorelapsed SCLC tumor tissue.

## Introduction

Small cell lung cancer (SCLC) is known as a highly aggressive type of cancer with a miserable prognosis. It is the second most common lung cancer type and accounts for ~15% of lung cancer cases. Therapy remained essentially unchanged in recent years and is usually based on chemotherapy with etoposide and a platinating agent (1), whereas, lately, clinical activity of immunotherapies has been observed in patients with refractory or metastatic SCLC. In a phase 3 trial conducted by Horn et al. it was shown that the addition of the anti PD-L1 antibody atezolizumab to chemotherapy in the first-line treatment of extensive SCLC resulted in significantly longer overall survival (OS) and progression-free survival than chemotherapy alone (2). Almost all patients experience disease relapse within 3 months (3). Although SCLC-targeted therapy research has progressed, in contrast to non-small cell lung cancer (NSCLC) no personalized targeted therapy options have been derived so far. Thus, further research into the mechanism of SCLC and the exploration of new therapeutic targets for SCLC are indispensable (4).

A new promising target is Delta-like protein 3 (DLL3), a transmembrane protein found in most high-grade neuroendocrine carcinomas of the lung, including SCLC. Growing evidence supports a tumor-suppressor role for Notch-1 signaling in neuroendocrine tumors (5).

It is shown that the NOTCH receptor is mainly downregulated by DLL3, thereby inhibiting the NOTCH signaling pathway within the cell. Inactivation of Notch directs the lung stem cell to a neuroendocrine precursor cell and contributes via biallelic p53/RB loss to the onset of primary SCLC (6). In this context, the achaete-scute homolog 1 (Ascl1) gene transcription factor plays a major role. It controls crucial cellular mechanisms in SCLC such as cell growth and survival. DLL3 expression is understood as a direct downstream target of Ascl1, which interacts with the DLL3 gene promoter (7). This suggests that, during evolution of SCLC, Notch1 is inactivated, and Ascl1 and DLL3 are both activated and are, thus, counterparts to Notch1.

Therefore, one can assume that DLL3 could also be used in cancer chemotherapy to target and suppress tumor cells (8). Rovalpituzumab tesirine (Rova-T) is an antibody–drug conjugate composed of SC16, a humanized IgG1 antibody against DLL3. Rova-T selectively binds to DLL3 on target-expressing cells, is internalized, and upon proteolytic cleavage, releases the toxin pyrrolobenzodiazepine (PBD) leading to cell death (9).

In a phase I clinical trial, Rova-T was more effective in SCLC with DLL3 overexpression (defined as expression in at least 50% of cancer cells by immunohistochemistry) compared with SCLC with a low level of DLL3 expression (5). Hence, DLL3 might be a promising predictive marker for treatment of SCLC with Rova-T (8). Rova-T is currently under development for patients with SCLC positive for DLL3 (3).

Methods to evaluate DLL3 expression assessed via immunohistochemistry are not standardized so far. In the current study, the two applied methods (tumor proportion score and H-score) were previously described in literature, including in the above mentioned trial (5, 10) and compared with each other.

Although most SCLC recur after initial response to chemotherapy, relapsed tumors are usually not biopsied. The aim of our study was, therefore, to investigate the expression of DLL3 on chemorelapsed SCLC samples and to compare its expression to chemonaive counterparts. Differences in DLL3 expression between matched samples would suggest therapy-associated changes in the tumor cells. In case of a loss of DLL3 expression in chemorelapsed SCLC samples one could assume that a therapy with Rova-T is not promising. The results of the study could be used to derive which material should be tested in case of planned therapy with Rova-T in the recurrence situation.

## Materials and Methods

### Cohort

In this multi-institutional retrospective study, tissue samples from chemonaive SCLC and paired recurrent SCLC after chemotherapy as well as from metastatic SCLC were collected. The final cohort included 42 patients and consisted of 30 paired chemonaive-chemorelapsed, 5 paired chemonaive primary-metastatic, and 7 unpaired chemorelapsed SCLC without a chemonaive counterpart. In the latter, from 3 patients, we had one sample each (only deriving from the primary site) and from 4 patients, we had several (up to 4) samples deriving from the primary site as well as from distant metastases (brain, lymph node, adrenal, liver, bone, contralateral lung). In the paired chemonaive-chemorelapsed subcohort, chemorelapsed tissue derived from local recurrences in 10 cases, from distant metastases in 19 cases (skin, brain, lymph node, bone, pleura, pericardium, breast, pancreas), and in one case, the location of the biopsied recurrent tumor was unknown. Chemonaive metastatic samples derived from adrenals, bone, liver, pleura, skin, and contralateral lung.

### Patient Characteristics

The median age in the whole cohort was 63 years (range, 47–83 y), 27 (64.3%) patients were male, 15 (35.7%), were female. All patients received a platin-based chemotherapy, whereas none of the patients had received therapy with Rova-T or other therapeutic agents for example in the context of an immunotherapy. Follow-up data for 33 out of the 42 patients were available. From these, at time of last follow-up, 19 patients were deceased, and 14 were alive. Samples from 4 patients derived from autopsies. The basic clinicopathological data of our study cohort are summarized in Table 1.

**Table 1:** Patients' baseline characteristics.

	**Total**	**Subcohort paired chemonaive-chemorelapsed**
**Patients**		
Male	27	20
Female	15	10
**Survival status**		
Alive	14	8
Deceased	19	15
Unknown	9	7
**Age at first diagnosis (years)**		
Mean	63.6	63.8
Median	63	63
Range	47–83	47–79
**Time span between diagnosis and last follow-up (days)**		
Min.	13	121
Max.	2.728	1.436
**Chemotherapy regime (partially known)**		
Cisplatin + etopside	14/23 (60.9%)	8/16 (50%)
Carboplatin + etopside	9/23 (39.1%)	8/16 (50%)
Mean duration (days)	87.2	90.9
Min. duration (days)	30	30
Max. duration (days)	284	284
Palliative intention	21/23	15/16 (94%)
Adjuvant intention	1/23	0
Neoadjuvant intention	1/23	1/16 (6%)
**Time span between chemonaive and chemorelapsed sample (days)**		
Min.		48
Max.		1.294
Mean		404.1
Median		249
**Time span between therapy initiation and relapse (days)**		
Min.	15	92
Max.	1.297	1.297
Mean	430.9	421.7
Median	333.5	248
**Tumor site**		
Primary tumor + recurrent tumor same site	14	10
Primary tumor + recurrent tumor different site	27	19
Unknown	1	1
**DLL3 expression (chemonaive vs. chemorelapsed sample)**		
TPS 0%		4 vs. 6
TPS < 1–49%		10 vs. 5
TPS ≥ 50%		16 vs. 19
H-Score 0		4 vs. 6
H-Score < 150		16 vs. 8
H-Score ≥ 150		10 vs. 16
**DLL3 expression dynamics**		
Stable expression		19 (63.3%) with TPS vs. 17 (56.6%) with H-Score
Higher expression in chemonaive sample		4 (13.3%) with TPS vs. 4 (13.3%) with H-Score
Higher expression in chemorelapsed sample		7 (23.3%) with TPS vs. 9 (30%) with H-Score

This study was approved by the internal review board of the University of Luebeck (file number 16-277) and the respective local ethical committees.

### Statistical Analyses

For the statistical analyses and data visualization, R software (version 4.0.2, R Foundation, Vienna, Austria; http://www.R-project.org) was used. To investigate differences of DLL3 expression between the group of chemonaive and chemorelapsed samples a Wilcoxon signed-rank test was used. To test if the two evaluation methods (TPS vs. H-score) give equivalent results, Pearson correlation was applied. The Wilcoxon rank-sum test was applied to correlate the site of the recurrent tumor and DLL3 expression. To analyze for correlation of DLL3 expression with clinicopathological characteristics, Fisher's exact test was used. Kaplan–Meier curves were used to illustrate OS in dependency of DLL3 expression and statistically proved by log-rank tests. A *p*-value of <0.05 was considered significant.

### Immunohistochemistry (IHC)

IHC staining was performed according to the manufacturer's instructions, using the Ventana Discovery (Ventana Medical System) automated staining system. In brief, slides were incubated with the primary antibody DLL3 (clone SP347, Ventana, SN 678, RTU). Representative tumor blocks from FFPE tissue were cut in 4-μm thick sections.

DLL3 staining was considered positive if staining was membranous in at least 1% of tumor cells. Protein expression of DLL3 was assessed in two different ways: on the one hand by estimating the percentage of positive tumor cells from all tumor cells (tumor proportion score, TPS, range 0–100%) as previously described (5) and, on the other hand, semi-quantitatively using the H-score method according to Yan et al. by converting the staining intensity (SI) (range 0–3) and the TPS (range 0–100) to a H-score (range, 0–300) (10). SI was indicated as strongly positive (SI 3), moderately positive (SI 2), weakly positive (SI 1) and negative (SI 0). To dichotomize samples into positive and negative staining, TPS < 50% and H-score ≤ 150 were defined as DLL3 low expression (DLL3-low), and TPS ≥ 50% and H-score > 150 were defined as DLL3 high expression (DLL3-high) (5, 10).

## Results

### Expression Pattern of DLL3 Protein in Unpaired Chemorelapsed SCLC Samples

In seven cases of the whole cohort, we had solely chemorelapsed samples without a chemonaive counterpart. Of these, from three patients, we had one sample each (only deriving from the primary site), and from four patients, we had several (up to four) samples deriving from the primary site as well as from distant metastases. From the three cases with one sample, two were DLL3-high and one was DLL3-low, assessed by TPS. From the four cases with several samples, three (75%) showed a concordant DLL3 expression between the samples (two DLL3-low, one DLL3-high), and the other one showed high-DLL3 expression in the primary tumor and low-DLL3 expression in the metastatic site. Considering only the staining result of the first sample of each patient, four were DLL3-high and three DLL3-low. One of the two cases with concordant DLL3-low expression derived from an autopsy so that loss of expression may originate from autolysis.

### Expression Pattern of DLL3 Protein in Paired Chemonaive Primary and Metastatic SCLC Samples

Three of the five cases with paired chemonaive primary and metastatic SCLC samples showed a concordant DLL3-high staining pattern, and in two cases, we observed a high-DLL3 expression in the primary tumor and a low expression in the metastatic site, assessed by TPS. One case derived from an autopsy, and here, we investigated four samples deriving from different sites (adrenal, bone, liver, contralateral lung), all showing concordant low DLL3 expression. Here, concordant low expression might also be due to autolytic changes of the tissue.

### Expression Pattern of DLL3 Protein in Paired Chemonaive-Chemorelapsed SCLC Samples

**Figure 1:**
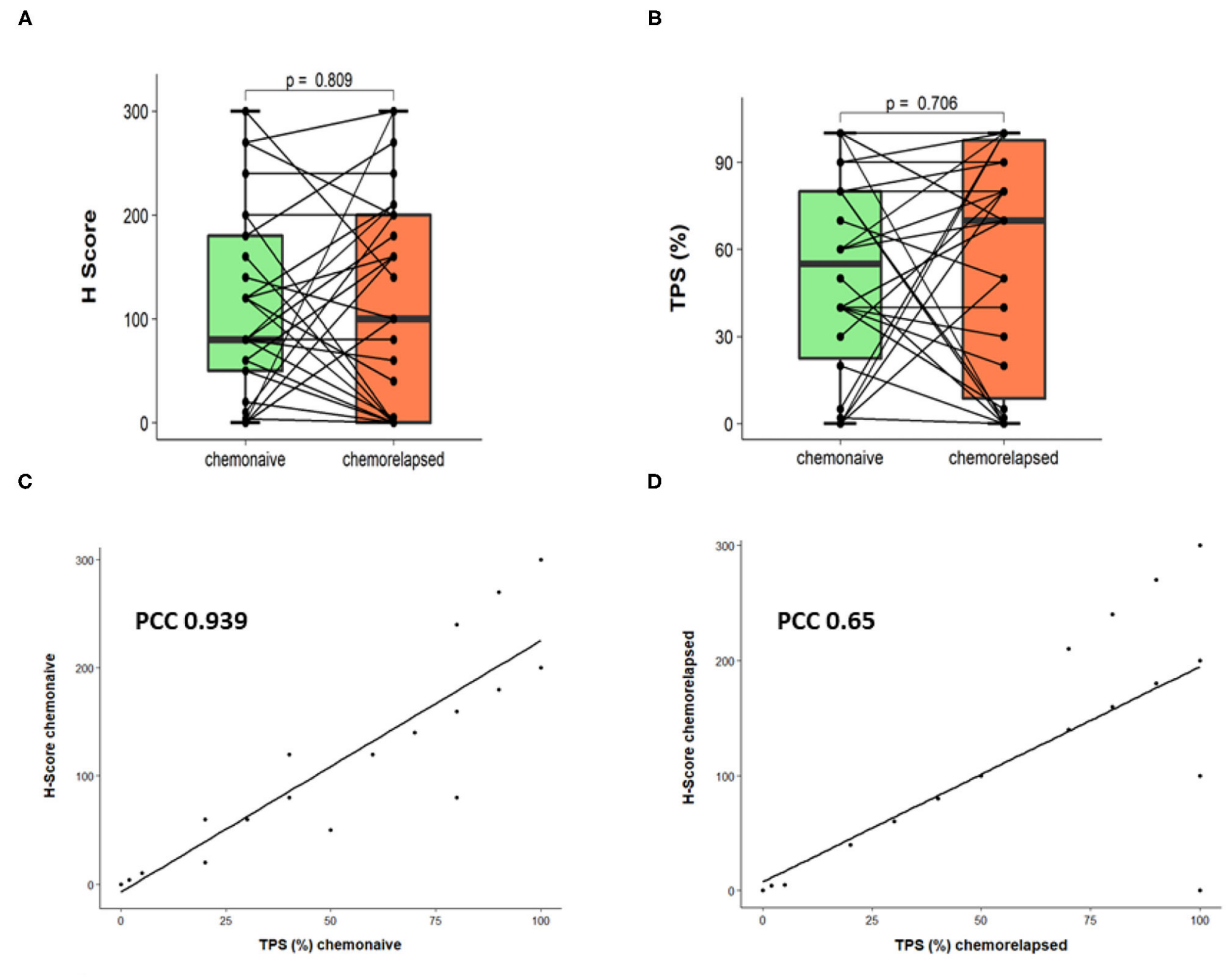
Comparison of DLL3 expression in paired chemonaive-chemorelapsed SCLC samples. **(A)** DLL3 expression assessed by H-score. **(B)** DLL3 expression assessed by TPS. **(C)** Pearson correlation showing application of TPS and H-score on chemonaive SCLC samples. **(D)** Pearson correlation showing application of TPS and H-score on chemorelapsed SCLC samples.

Considering only the chemonaive group, using the H-score method, 20 (66.6 %) out of 30 samples were DLL3-low, and 10 (33.3%) were DLL3-high. Out of the DLL3-low samples, four were negative (H-score 0). In the chemorelapsed group, 14 (46.6 %) were DLL3-low and 16 (53.3%) were DLL3-high. Here, out of the DLL3-low samples, 6 were negative (H-score 0). DLL3 expression between the chemonaive and chemorelapsed groups was not significantly different (Wilcoxon signed-rank test *p* = 0.809; Figure 1A). Concordant staining, either DLL3-low or DLL3-high in paired chemonaive-chemorelapsed specimens, was observed in 17 out of 30 (56.6%) SCLCs. From those, in 11 cases (64.7%), both samples were DLL3-low, and 6 (35.9%) were DLL3-high. In 13 cases (43.4%) staining was not concordant, meaning that, in 9 cases, the chemonaive sample was DLL3-low and the chemorelapsed sample was DLL3-high (“DLL3 up”) and that in 4 cases, the chemonaive sample was DLL3-high, and the chemorelapsed sample was DLL3-low (“DLL3 down”).

**Figure 2:**
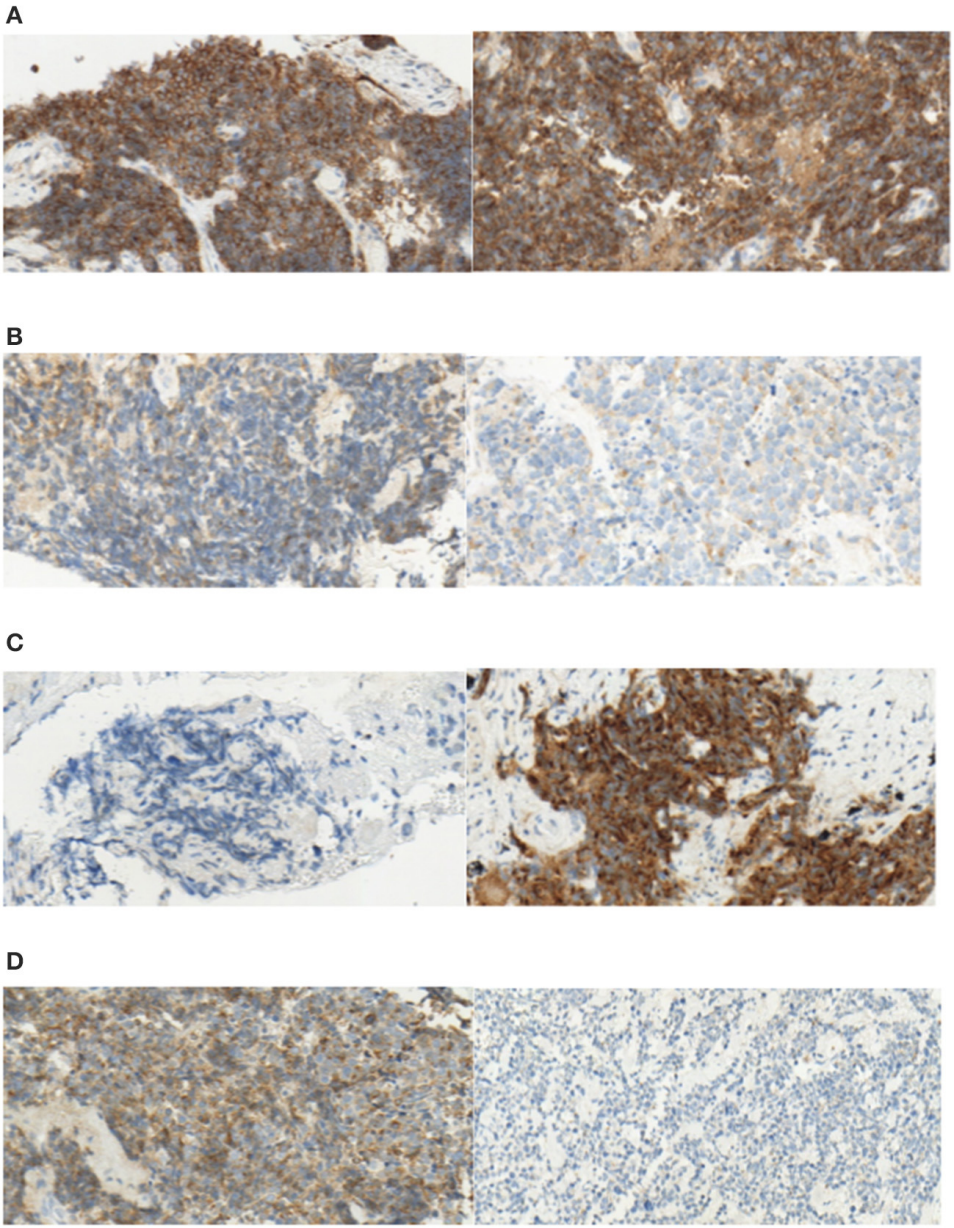
Exemplary pictures of DLL3 staining patterns in paired chemonaive (left) and chemorelapsed (right) SCLC samples. **(A)** Both high DLL3 expression (TPS > 50%, H-score > 150), **(B)** Both low DLL3 expression (TPS < 50%, H-score < 150), **(C)** “DLL3 up” [low expression in chemonaive sample (TPS < 50%, H-score < 150) and high expression in chemorelapsed sample (TPS > 50%, H-score > 150)], **(D)** “DLL3 down” [low expression in chemorelapsed sample (TPS < 50%, H-score < 150) and high expression in chemonaive sample (TPS > 50%, H-score > 150); original magnification ×40].

With regard to TPS, 14 (46.6%) chemonaive samples were DLL3-low, and 16 (53.4%) were DLL3-high. In the chemorelapsed group, 11 (36.6%) samples were DLL3-low and 19 (63.3%) were DLL3-high. Negative samples (TPS < 1%) within DLL3-low samples were identical to evaluation with H-score (4 in the chemonaive and 6 in the chemorelapsed groups, respectively). Again, DLL3 expression between the chemonaive and chemorelapsed group was not significantly different (Wilcoxon signed-rank test *p* = 0.706; Figure 1B). In the chemonaive group, mean TPS was 52.2%, ranging from 0 to 100%. In the chemorelapsed group, mean TPS was 57.6%, ranging from 0 to 100%. With regard to expression dynamics, 7 out of 30 samples (23.3%) showed equally low DLL3 expression, and 12 (40%) showed equally high expression, meaning that, in 63.3%, TPS was stable between chemonaive and chemorelapsed samples. In 36.7% (*n* = 11), DLL3 expression between the matched samples was different. In 7 cases (23.3%), the chemorelapsed sample had a high TPS, and the chemonaive counterpart a low TPS (DLL3 up), and in 4 cases (13.3%) the dynamic was the other way around (DLL3 down). DLL3 expression data are summarized in Table 1, and representative images of DLL3 protein expression assessed by IHC are provided in Figure 2.

Two of the cases originated from autopsies. Of these, one case showed a DLL3-down expression pattern assessed with the H-score method as well as with TPS. Again, this could be due to autolytic changes of the tissue. However, the other case showed an equally low expression without the chemorelapsed sample being completely negative (TPS 20%, SI2). Therefore, it cannot be stated in general that DLL3 expression is lost in autopsy material.

To assess both evaluation methods, we correlated TPS and H-score. Pearson correlation provided a linear dependency (PCC 0.939 for chemonaive samples, PCC 0.65 for chemorelapsed samples, Figures 1C,D), which proved that both methods were equally applicable. Because the TPS is easier to use in everyday diagnostic practice and probably shows less interobserver variability, only TPS was used for further statistical analysis.

**Figure 3:**
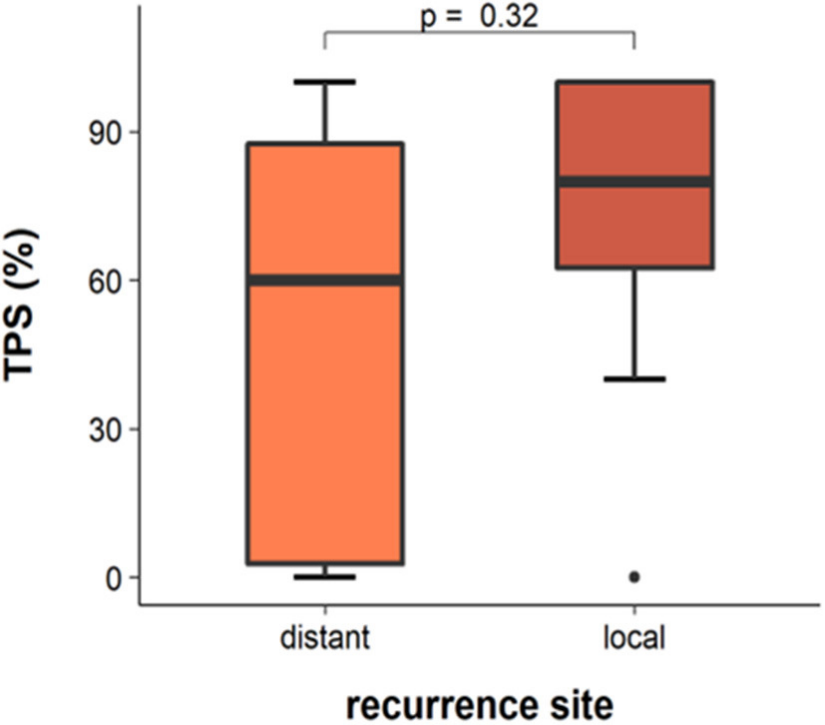
DLL3 expression in chemorelapsed samples in dependency of site of recurrent tumor.

We next evaluated if there was a correlation between tumor site and DLL3 expression. We found no significant differences between local pulmonal and distant recurrences (Wilcoxon rank-sum test *p* = 0.32; Figure 3).

### Correlation of DLL3 With Clinicopathological Characteristics in the Paired Chemonaive-Chemorelapsed SCLC Cohort

There was no significant correlation of DLL3 expression level estimated in chemonaive samples with regard to gender, T-status, N-status, M-status, UICC-status, or time to recurrence. The only significant result was shown with regard to age: DLL3-high SCLCs were more frequent in younger patients (median age 57 years) and SCLC with low DLL3 expression more frequent in older patients (median age 67) (*p* = 0.024) (Table 2). We performed the same analysis for the DLL3 expression level estimated in chemorelapsed samples and, however, found no significant result for any of the above mentioned parameters (not shown).

**Table 2:** Association analysis between DLL3 expression assessed in chemonaive samples and clinicopathological characteristics of paired chemonaive-chemorelapsed SCLC cohort.

**DLL3 expression assessed in chemonaive sample**	**Total (*n* = 30)**	**TPS ≧ 50% (*n* = 16)**	**TPS < 50% (*n* = 14)**	***p*-value**
**Gender**				0.26
Male	20 (66.7%)	9 (56.2%)	11 (78.6%)	
Female	10 (33.3%)	7 (43.8%)	3 (21.4%)	
**Age**				0.024
Missing	1	1	0	
Median (IQR)	65 (57, 70)	57 (55, 68)	67 (61, 75)	
**T-Stage**				0.58
Missing	16	8	8	
T(1, 2)	4 (28.6%)	3 (37.5%)	1 (16.7%)	
T(3,4)	10 (71.4%)	5 (62.5%)	5 (83.3%)	
**N-Status**				1
Missing	16	8	8	
N–	2 (14.3%)	1 (12.5%)	1 (16.7%)	
N+	12 (85.7%)	7 (87.5%)	5 (83.3%)	
**M-Status**				1
Missing	16	8	8	
M–	4 (28.6%)	2 (25.0%)	2 (33.3%)	
M+	10 (71.4%)	6 (75.0%)	4 (66.7%)	
**UICC-Stage**				1
Missing	16	8	8	
< IV	4 (28.6%)	2 (25.0%)	2 (33.3%)	
IV	10 (71.4%)	6 (75.0%)	4 (66.7%)	
**Time to recurrence (months)**				0.693
Missing		1	0	
Mean (SD)	12.8 (11.6)	10.4 (7.6)	15.4 (14.5)	
Median (IQR)	8 (5, 16)	8 (6, 14)	8.5 (4, 23.5)	

### Correlation of DLL3 With OS in the Paired Chemonaive-Chemorelapsed SCLC Cohort

The median age of this subcohort was 63 years (range, 47–79), 20 (66.6%) were male, 10 (33.3%), were female. From 23 out of the 30 patients, data concerning survival status was available. The median follow-up time for the patients with SCLCs was 354 days (range, 121–1,436). At the time of last follow-up, 15 patients were deceased and eight were alive.

**Figure 4:**
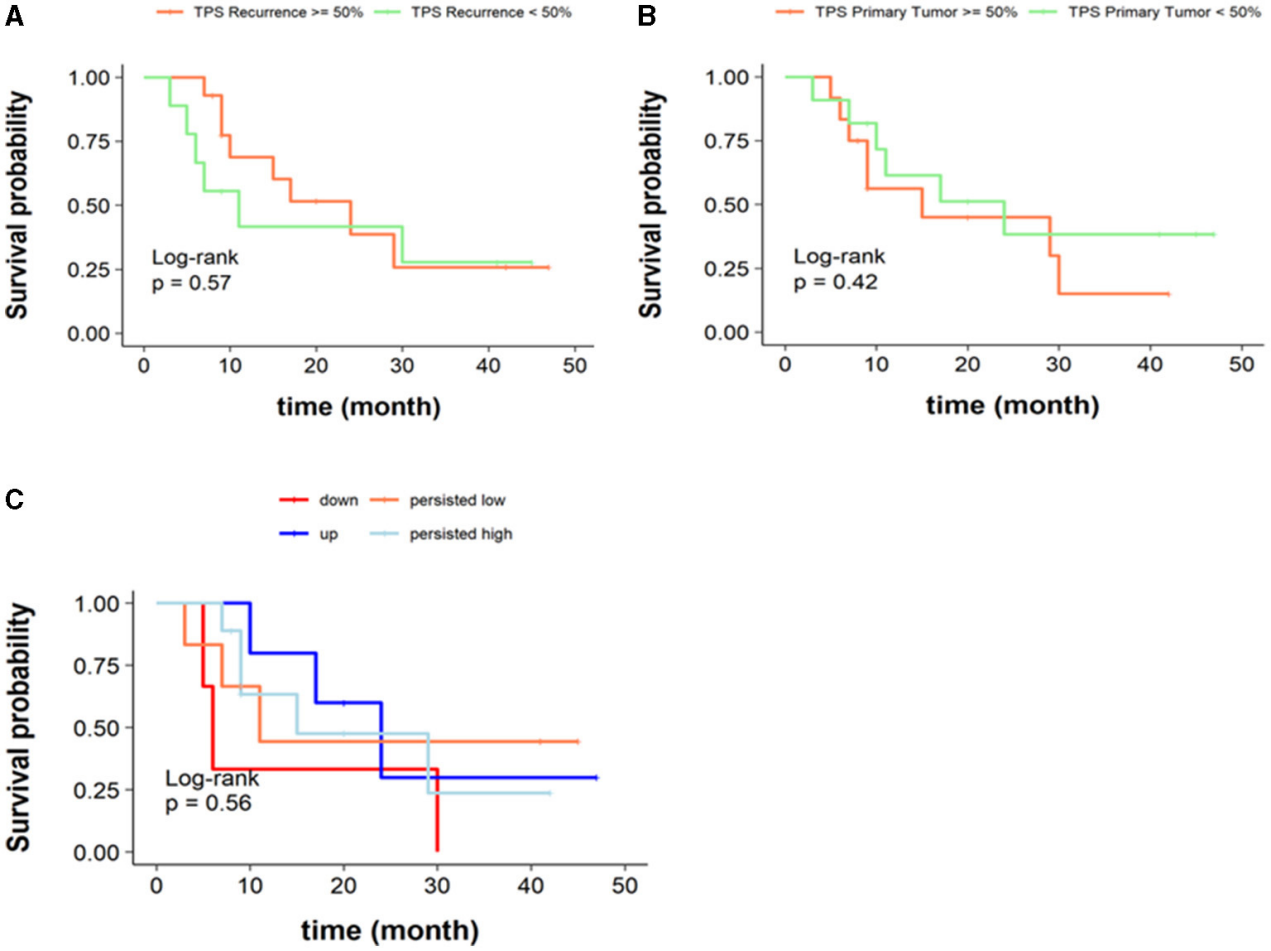
Overall survival according to DLL3 expression status. **(A)** Kaplan–Meier curves stratified according to DLL3-high or DLL3-low status assessed with TPS in chemorelapsed specimens (log-rank *p* = 0.57). **(B)** Kaplan–Meier curves stratified according to DLL3-high or DLL3-low status assessed with TPS in chemonaive specimens (log-rank *p* = 0.42). **(C)** Kaplan–Meier curves stratified according to dynamics of DLL3 expression in the course of therapy. Persisted DLL3-low was defined as TPS < 50% in chemonaive and chemorelapsed samples, persisted DLL3-high was defined as TPS ≥ 50% in chemonaive and chemorelapsed samples, “up” was defined as a switch from TPS < 50% to ≥ 50%, and “down” was defined as a switch from TPS ≥ 50% to <50% in the course of therapy (log-rank test *p* = 0.56).

We analyzed whether DLL3 expression (TPS low vs. high) in chemorelapsed or chemonaive samples could predict OS. Although results are not significant, Kaplan–Meier curves show a trend for better OS if DLL is highly expressed in chemorelapsed samples (log-rank *p* = 0.57) and less expressed in chemonaive samples (log-rank *p* = 0.42) (Figures 4A,B), whereas a low DLL3 status in chemorelapsed samples and a high DLL3 expression status in chemonaive samples seem to be more unfavorable. Next, we aimed to analyze if the dynamics of DLL3 expression during therapy has an impact on OS. As the above mentioned results already suggest, upregulation of DLL3 expression in matched samples after therapy reveals a trend for a better OS, whereas the worst survival rates were seen in cases with a downregulated DLL3 expression (log-rank test *p* = 0.56; Figure 4C). Survival curves indicating cases in which DLL3 expression stayed stable, either both low or high, lay in between the two curves indicating an upregulated or downregulated DLL3-status and show also a trend for a more favorable survival in high DLL3 expressing tumors.

## Discussion

SCLC is one of the most aggressive tumors with so far very limited therapeutic options (5, 9). Essentially all patients with extensive-stage SCLC and the majority of patients with limited-stage SCLC suffer relapse within months of completing initial standard therapy (11). As SCLC is rarely biopsied following the initial diagnosis, dynamics of expression of therapeutically relevant biomarkers in relapsed disease are poorly understood. Because DLL3 is largely expressed in SCLC and regarded as a potential biomarker for response to Rova-T treatment (8), we aimed to investigate DLL3 expression in chemorelapsed SCLC samples and to compare its expression with matched chemonaive SCLC samples, correlate its expression with clinicopathological data, and perform survival analysis stratified according to DLL3-high or DLL3-low status and dynamics of DLL3 expression during the course of therapy. Possible differences in expression may further indicate if chemonaive or chemorelapsed SCLC specimens should be investigated in cases of a planned therapy with Rova-T. To our knowledge, there are hardly any published studies that investigated DLL3 expression in chemorelapsed SCLC samples and paired chemonaive-chemorelapsed SCLC samples, respectively.

In line with data from the literature, we as well found DLL3 expression in the majority of our SCLC samples. The evaluation method to assess DLL3 expression is not standardized, and there are so far no international standards for cut-off values to determine expression of DLL3 (4).

When comparing the DLL3 expression data of our study with those from the literature, it is important to consider not only the evaluation method, but also the diversity of the DLL3 antibodies used. Brcic et al. (12) investigated four different DLL3 antibodies (VenA (clone SP347; Ventana, Roche, Tucson, AZ, USA), NovA (NBP2–24669; Novus Biological, Littleton, CA, USA), TherA (PA5–26336; Thermo Fisher Scientific, Waltham, MA, USA), and AbcA (ab103102; Abcam, Cambridge, MA, USA)) for their reliability to detect DLL3 expression in high-grade neuroendocrine tumors of the lung. Comparison of VenA [the antibody used in the current study and in the clinical trial (5)] with the other three antibodies demonstrated poor results for overall agreement, positive and negative agreement, and Kappa values. The authors concluded that using VenA as a reference antibody, none of the other three antibodies can reliably be used for the DLL3 test. This, of course, makes comparison of DLL3 expression data between studies difficult. Table 3 gives an overview of some recent studies dealing with DLL3 expression in SCLC, including their cohort sizes, evaluation methods, used DLL3 antibodies, used cutoffs, and assessed expression data of DLL3 in SCLC.

**Table 3:** Overview of recent studies on DLL3 expression in SCLC.

**Study**	**Tissue related to therapy**	**Cohort size (*n*)**	**DLL3 antibody**	**Evaluation method**	**Cutoff**	**DLL 3 expression**
Yan et al. (10)	Chemonaive (“*de novo”*)	335	ab103102; Abcam, Cambridge, UK	H-Score	≤ 150 low vs. > 150 high	37.6% low (126/335)
						62.4% high (209/335)
Tanaka et al. (3)	not applicable	63	Stemcentrx, South San Francisco, CA, USA	TPS	<1%, 1–49%, ≥50%	<1%: 17% negative (11/63)
	Presumably Chemonaive					1–49%: 51% (32/63)
						≥50%: 32% (20/63)
Xie et al. (13)	not applicable	44	Clone SP346, Ventana-Roche Diagnostics, Indianapolis, IN, USA	TPS plus four-level SI, but cut-off based on TPS	<50% low, ≥50% high	20.5% low (9/44)
						79.5% high (35/44)
Regzedmaa et al. (8)	Chemonaive	38	No SAB1302862, Sigma-Aldrich, Shanghai, China	TPS scored as 1 (≤ 25%), 2 (26–50%), 3 (51–75%), 4 (>75%) multiplied with four-level SI	Median	47.4% low (18/38)
						52.6% high (20/38)
Huang et al. (14)	Chemonaive (“firstly diagnosed”)	72	ab103102, Abcam, Cambridge, MA, USA	TPS scored as 1 (1–9%), 2 (10–49%), 3 (50–79%), 4 (≥80%) multiplied with four-level SI	<6 low, ≥6 high	68% low (49/72)
						32% high (23/72)
Furata et al. (15)	Chemonaive (“primary”)	93	SP347; Spring Bioscience, Pleasanton, CA	TPS	<75% low, ≥75% high	53% low (49/93)
						47% high (44/93)
Rudin et al. (5)	not applicable	39	Stemcentrx, South San Francisco, CA, USA	TPS	<50% low, ≥50% high	25.6% low (10/39)
						74.4% high (29/39)
Rojo et al. (16)	Diverse (independent and paired)	1,050	Clone SP347, Ventana, Tucson	TPS Negative (0–24%), positive (≥25%), non-high positive (25–74%), high positive (≥75%)	Positive (≥25 %)	15% negative (155/1050)
						85% positive (895/1050)

We assessed DLL3 expression in two different ways, using the H-score and TPS, both known from prior studies (5, 10). We adopted 50% as the cutoff for TPS from a phase I trial conducted by Rudin et al. (5) and, with that, found expression of DLL3 in majority of chemonaive samples (86.6%) out of which 46.6% were DLL3-low and 53.4% DLL3-high. In the study from Rudin et al. (5), the range was higher with 74.4% being highly positive (29/39) and 25.6% (10/39) being weakly positive. The same cutoff was used by Tanaka et al., who investigated 63 presumably chemonaive SCLC samples and found 83% (52/63) positive for DLL3 with 20 samples (32%) being highly positive (3). The data in the literature vary; nevertheless, overall, a quite high rate of highly DLL3 expressing SCLC can be observed. In the group of chemorelapsed SCLC, we found similar DLL3 expression to that in the chemonaive group with 80% being positive, but with a slightly higher proportion of DLL3-high cases (63.3%, 19/30). DLL3 expression between chemonaive and chemorelapsed was stable in more than half of the cases (63.3%) and shifted from low to high or vice versa in 36.7%. However, DLL3 expression between chemonaive and chemorelapsed SCLC samples was not significantly different (Wilcoxon signed-rank test *p* = 0.706; Figure 1B) indicating no essential therapy-induced differences.

For evaluation with H-score, we adopted the cutoff of 150 from Yan et al. (10), who investigated 335 presumably chemonaive (*de novo*) SCLC samples and found a low expression (H-score ≤ 150) in 37.6% and a high expression (H-score > 150) in 62.4%. We found contrasting results with this method with a low expression in 66.6% (20/30) in the chemonaive group and a high expression in 33.3% (10/30) in the chemorelapsed group. In the chemorelapsed group instead, expression data were then again similar to those from above mentioned study with a shift to more highly positive SCLC cases [(53.3%, 16/30) vs. 46.6% (14/30) DLL3 low expressing samples]. However, the data are hardly comparable due to the significant differences in the size of the cohorts (335 vs. 30).

Concordant staining in paired chemonaive-chemorelapsed specimens, either both DLL3-low or DLL3-high, was found in only approximately half of the cases (56.6%), meaning that a significant portion (43.4%) showed a shift of DLL3 expression during course of therapy. However, also with this method, we found no significantly different DLL3 expressions between the two groups (Wilcoxon signed-rank test *p* = 0.809; Figure 1A).

With both evaluation methods, the proportion of matched samples showing deviating DLL3 expression, meaning a shift from low to high expression or vice versa, during course of therapy was considerable (43.4% with H-score, 36.7% with TPS). This indicates that DLL3 expression might be influenced by therapy and cannot be considered as stable. Unlike other predictive biomarkers, such as, e.g., PD-L1 with a required TPS of 50% for therapy of NSCLC with Pembrolizumab, so far, a high (≥50%) DLL3 expression of SCLC is not implemented as a prerequisite for therapy with Rova-T. However, if this were the case, our data demonstrate that it does matter which samples are chosen to assess DLL3 expression. There is only a little literature concerning DLL3 expression in paired chemonaive-chemorelapsed SCLC samples. Rojo et al. (16) investigated, in addition to a huge number of independent SCLC samples, also 36 paired SCLC samples, defining “paired” as two specimens from the same patient and same primary disease site or as a first specimen obtained at diagnosis and the second obtained at relapse/recurrence. Of these, only two samples corresponded to paired chemonaive-chemorelapsed samples as we had examined. Moreover, they used a different cutoff than we did with ≥25% positive tumor cells defined as positive DLL3 expression (Table 3). With that, they found 88% concordance between paired specimens without specifically addressing the paired chemonaive-chemorelapsed samples. Due to the significant deviation in size of the cohorts, results of this study are hardly comparable with our results.

To our knowledge, the above mentioned study by Yan et al. (10) is the only one using the H-score method with a cutoff of 150. Furthermore, combining a four-level SI and TPS to a H-score should show higher interobserver variability than just using TPS. We, therefore, assessed the two evaluation methods, and after Pearson correlation provided a linear dependency that indicated equality, we have then focused on TPS for further analyses.

We next investigated if the site of the biopsied recurrent tumor has an impact on DLL3 expression. In 33.3% (10/30) chemorelapsed tissue derived from local pulmonal recurrences and in 63.3% (19/30) from distant metastases. Concordant with the literature (3, 10), we found no significant differences of DLL3 expression between local pulmonal and distant recurrences (Wilcoxon rank-sum test *p* = 0.32; Figure 3). Yan et al., for instance, compared intertumoral expression of DLL3 on the basis of 37 paired biopsies of primary and metastatic sites and found concordant staining in all cases (10).

Apart from age when considering the chemonaive samples, we found no association of DLL3 expression with clinicopathological data in our cohort (Table 2). Our observation that DLL3 expression actually does not associate with clinicopathological data broadly fit with those from the literature. For instance, Tanaka et al. (3) also found no significant association between DLL3 expression and age, sex, smoking history, or disease stage, and Yan et al. (10) found no significant association with age, distant metastasis status, or TNM stage. In the latter study, DLL3 expression was higher in TTF-1 expressing SCLC samples (*p* = 0.006), smokers (*p* = 0.023), and males (*p* = 0.041), whereas high DLL3 expression was associated with female sex (*p* = 0.03) in a study conducted by Xie et al. (13). Furata et al. found that DLL3-high expression (defined as TPS ≥ 75%) was significantly more prevalent in patients with lymph node metastases and advanced c-stage (15). These few significant correlations found in our study and in the literature are, due to their diversity, primarily a coincidence and might not have a causal relationship.

We next investigated if DLL3 expression has an impact on OS. Kaplan–Meier curves do not show significant results, but a trend for a better OS if DLL is highly expressed in chemorelapsed samples (log-rank *p* = 0.57) and low expressed in chemonaive samples (log-rank *p* = 0.42) (Figures 4A,B), whereas a low DLL3 status in chemorelapsed samples and a high DLL3 expression status in chemonaive samples seem to be more unfavorable. Several other studies also analyzed the relationship of DLL3 expression and OS, and the corresponding results are partly contradictory. Some studies found no statistically significant difference of OS between DLL3 low and high expressing tumors (3, 15), whereas Regzedmaa et al. found that high expression of DLL3 assessed on chemonaive SCLC correlated significantly with poorer patient outcomes (*p* = <0.001) (8). Yan et al. also found that patients with chemonaive SCLC having a high DLL3 expression level exhibited a lower OS compared with patients with DLL3-low expressing SCLC (*p* = 0.007). In this study, expression of DLL3 and TTF-1 was investigated in combination, and additionally, it was found that the group of SCLC with low expression of DLL3 in combination with missing TTF-1 expression showed improved OS (10). Huang et al. also found a high level of DLL3 to be correlated with low OS rate (*p* < 0.01) (14). Despite lacking significance, our survival curves also indicate that there is a trend for better survival if chemonaive samples show low DLL3 expression (Figure 4B). Xie et al. found that high DLL3 expression was associated with better OS in SCLC (*p* = 0.049). At first glance, the results seem contradictory. However, in this study, only a few cases showed low DLL3 expression, and after adjusting for age, tumor size, and stage, DLL3 expression was no longer associated with OS (13).

Due to our samples being matched, we then analyzed if the dynamics of DLL3 expression in the course of therapy associates with survival. As the above mentioned results already suggest, upregulation of DLL3 expression in matched samples after therapy reveals a trend for better overall survival, whereas the worst survival rates were seen in cases with a downregulated DLL3 expression (log-rank test *p* = 0.56; Figure 4C). Survival curves indicating cases in which DLL3 expression was concordant in chemonaive and chemorelapsed samples, either both low or high, lay in between the two curves, indicating survival in dependency of an upregulated or downregulated DLL3-status. Here, a trend that stable high expression is associated with a more favorable survival than stable low expression can be derived. This might suggest that DLL3 expression assessed in chemorelapsed samples is more meaningful regarding OS due to beforehand we could show that a low expression in chemonaive samples and a high expression of DLL3 only in chemorelapsed samples indicated a better OS (Figures 4A,B).

In a study from Tendler et al. (7), it was found that subjects with Notch1 low expressing chemorelapsed SCLC samples showed a better prognosis and higher sensitivity to chemotherapy. Assuming that Notch1 and DLL3 are opponents during the evolution of SCLC, one could expect a high DLL3 expression if Notch 1 shows a low expression. Thus, our result for a trend for better prognosis in DLL3 high expressing chemorelapsed SCLC is in line with that of Tendler et al.

To our knowledge, our study is the first investigating DLL3 expression in a large cohort of paired chemonaive-chemorelapsed SCLC samples, which is why our results concerning OS in relation to the dynamics of DLL3 expression cannot be compared with data in the literature. Our survival data is not significant. This might be due to the relatively small cohort size, which, at the same time, represents a limitation of our study. However, against the background that, in most cases, recurrent SCLC is not biopsied at all, our cohort is exceptional. Another limitation of our study is that recent literature (17, 18) on DLL3 seems to diminish its value as a potential therapeutic agent, which also weakens the value of the present work.

To sum up, the current study delivers, for the first time, data concerning DLL3 expression in a large cohort of rare paired chemonaive and chemorelapsed SCLC samples, which is exceptional due to relapsed SCLC are usually not being biopsied. However, investigation of chemorelapsed tumor tissue is essential because it might provide hints why early recurrences, characteristic for SCLC, occur. As the first, we show that, in a large proportion of paired chemonaive-chemorelapsed SCLC samples, DLL3 expression is not stable during the course of therapy, indicating therapy-associated alterations. This demonstrates that it is worth assessing protein expression of biomarkers in general and here, especially of DLL3 in chemorelapsed samples, meaning in the latest tumor tissue. This should, at the same time, be an incentive to gain tumor tissue by biopsy in relapses. Due to the manner of assessing expression of DLL3 not being standardized, we tested two evaluation methods and applied TPS and H-score. Both approaches delivered comparable results, but because TPS should be more practical in routine diagnostics than the H-score method, we have continued working with TPS. As in other studies, we found that the majority of SCLC samples expressed DLL3 and also did not find any profound correlations with clinicopathological data or tumor site. Concordant with data from the literature, our survival analysis revealed a trend for a better OS if DLL is low expressed (<50%) in chemonaive samples. Interestingly, this does not seem to apply to chemorelapsed SCLC because, for those, we could observe a trend for a more favorable survival in the event of a high expression (≥50%). In line with that, cases showing a shift from a low to a high expression of DLL3 during the course of therapy indicated the most favorable survival data. These results should not be overstated due to the size of our cohort not being powered to detect association with survival data. However, the study definitely shows the importance of research of chemorelapsed tumor tissue.

## Data Availability Statement

The raw data supporting the conclusions of this article will be made available by the authors, without undue reservation.

## Author Contributions

SP and CK planned the research project and evaluated the samples. ED performed the immunohistochemical stainings. TJ performed the statistical analysis. F-OP, CH, BK, SBo, BM, MA, SBe, MT, HB, and FF provided patients‘ follow-up data. CK, TJ, RK, F-OP, CH, BM, JR-I, CI, MA, SBe, MT, HB, FF, BK, SBo, VS, JK, and SP wrote and/or revised the manuscript. All authors have read and agreed to the published version of the manuscript.

## Funding

This research was supported with funds from the Section of Medicine at the University of Luebeck J18-2020.

## Conflict of Interest

SP is a consultant of Ventana, Roche, Novartis, Astellar, Astrazeneca, Bristol-Myers Squibb, Merck Serono and MSD. JK is a consultant of Roche, Novartis, BMS and MSD. BM received an honorary from AbbVie for a one-time consulting activity. The remaining authors declare that the research was conducted in the absence of any commercial or financial relationships that could be construed as a potential conflict of interest.

## Publisher's Note

All claims expressed in this article are solely those of the authors and do not necessarily represent those of their affiliated organizations, or those of the publisher, the editors and the reviewers. Any product that may be evaluated in this article, or claim that may be made by its manufacturer, is not guaranteed or endorsed by the publisher.

## Abbreviations

Ascl1: Achaete-scute homolog 1
DLL3: Delta-like protein 3
IHC: Immunohistochemistry
NSCLC: Non-small cell lung cancer
OS: overall survival
Rova-T: Rovalpituzumab tesirine
SCLC: Small cell lung cancer
SI: staining intensity
TPS: Tumor proportion score.
